# A meta-analysis of the association of serum ischaemia-modified albumin levels with human hypothyroidism and hyperthyroidism

**DOI:** 10.1042/BSR20160268

**Published:** 2017-01-27

**Authors:** Varikasuvu Seshadri Reddy, Suman Bukke, Khageshwar Mahato, Vinod Kumar, Netala Vasudeva Reddy, Manne Munikumar, Bramahanapally Vodelu

**Affiliations:** 1 Department of Biochemistry, Maheshwara Medical College & Hospital, Chitkul, Patancheru, Telangana-502307, India; 2 Department of Biochemistry, Sri Venkateswara University, Andhra Pradesh, India; 3 Department of Biochemistry, Lady Hardinge Medical College, New Delhi, India; 4 Department of Biochemistry, BPS Government Medical College, Haryana, India; 5 Department of Biotechnology, Sri Venkateswara University, Andhra Pradesh, India; 6 Biomedical Informatics Center, National Institute of Nutrition, Telangana, India

**Keywords:** Ischemia-modified albumin, Oxidative stress, Thyroid dysfunction, Hypothyroidism, Hyperthyroidism, Meta-analysis

## Abstract

Serum levels of ischaemia-modified albumin (IMA) have been studied as a novel and simple measure of oxidative stress (OXS) in different thyroid pathologies. However, results of available studies in the literature were not consistent. This meta-analysis was attempted to quantify the overall effect size for serum IMA levels in human hypothyroidism (HT) and hyperthyroidism (HYT) and to study its associations with the thyroid profile. Databases of PubMed/Medline, EMBASE, Google Scholar, Web of Science and Science Direct were searched for articles. Data on serum IMA levels in HT, HYT patients and euthyroid controls were extracted to compute standardized mean differences (SMD) by the random-effects model. The associations between IMA and thyroid profile were computed by the meta-analysis of correlation coefficients. IMA levels in HT patients (SMD=1.12; *Z*=2.76; *P*=0.006) and HYT patients (SMD=1.64; *Z*=2.57; *P*=0.01) were significantly higher than in euthyroid controls and the thyroid treatment showed a favourble effect on serum IMA levels. There were strong and significant correlations between IMA and hormonal status in HT and HYT groups. This meta-analysis showing increased IMA level in both HT and HYT patients and its association with thyroid profile suggests that serum IMA could be used as a simple measure of increased OXS in thyroid dysfunction.

## Introduction

Thyroid disorders are majorly studied under two types: hypothyroidism (HT) and hyperthyroidism (HYT). HT is characterized by excess thyrotropin (TSH) and underproduction of thyroid hormones, whereas HYT is due to low TSH and overproduction of thyroid hormones. Both of these disorders can exist in clinical and subclinical states. While subclinical hypothyroid (SHT) patients show high TSH with normal thyroid hormone levels, subclinical hyperthyroidism (SHYT) is defined as having a low TSH level but normal thyroid hormones [1]. Thyroid diseases mentioned throughout the manuscript are either HT or HYT, unless specified as overt and/or subclinical type.

Our recent research [2,3] and previous literature [4–7] provide enough of evidence suggestive of oxidative stress (OXS) in association with thyroid disorders. OXS is defined as a dyshomoeostasis between free radical formation and counteraction. Excess free radical generation may cause structural and functional modifications to proteins, such as human serum albumin (HSA). OXS causes modification of the normal HSA at its metal-binding sites, decreasing its binding capacity for metals such as cobalt. This modified form of albumin is known as ischaemia-modified albumin (IMA) and its formation was first detected and reported as a promising marker for cardiac ischaemia [8]. However, IMA has been shown to be less specific for cardiac ischaemia due to its elevation in several non-cardiac diseases [9–11]. Recently, IMA has been studied as a novel, simple and promising marker of OXS in several endocrine diseases [12–14]. Literatures [15–23], including our recent report [23] have shown that high IMA levels were associated with OXS in thyroid disorders. However, these data concerning the serum IMA levels in thyroid dysfunction are limited and not consistent.

There are conflicting studies reporting a significant increase [15,19,23], decrease [20] and also reports where no change [17] in IMA was reported in overt hypothyroid (OHT) patients when compared with controls. There are also studies reporting contrast findings with a significant increase [18,23] and no change [17] in serum IMA level among SHT patients in comparison with euthyroid controls. Furthermore, some [19,21,22] but not all [16,20] studies have shown that IMA levels in the serum of overt hyperthyroid (OHYT) patients were different from euthyroid healthy controls. To the best of our knowledge, one study had reported no change in serum IMA in SHYT patients compared with controls [16], and also there were very few studies to include pre-treatment and post-treatment study design, reporting serum IMA level following thyroid therapy [16,19].

With this background of inconsistent evidence on serum IMA in thyroid dysfunction, in this meta-analysis, our objective was to test the hypothesis that serum IMA levels were different in thyroid patients (HT and HYT) compared with euthyroid controls. We, therefore, included all relevant publications to generate a quantitative overall effect size for serum IMA levels in human HT and HYT. Importantly, we have also performed the meta-analysis of correlations between IMA and thyroid profiles.

## Materials and methods

### Data sources and search strategy

Identification of relevant publications was performed in the NCBI PubMed database for the term IMA with the following: thyroid, thyroid disorder, thyroid disease, thyroidism, HT, HYT, OHT, SHT, OHYT and SHYT. Also to retrieve articles that were not indexed in the pubmed database, web of science, google scholar, Cochrane library, Springer’s author mapper and science direct databases were searched for studies that reported circulating IMA level in thyroid patients. Further, bibliographies of published articles were manually reviewed to identify additional studies. Three authors independently performed a literature search and any discrepancies were resolved by discussion.

All searches were carried out prior to June 23, 2016, with no time span specified, with humans set as a limit, with and without any language restriction. The literature retrieved was manually reviewed and the cited references were hand-searched for further identification of relevant studies. Efforts have been taken to obtain data, if any, from the unpublished sources. When required, the corresponding authors of respective articles were contacted through e-mail to obtain clarification.

### Study selection

Eligible studies had to meet the following criteria: (1) studies were published in a peer-reviewed journal; (2) studies provided original data; (3) study subjects were human adults with description of patients and controls; (4) studies where circulating levels of IMA in HT and/or HYT patients (overt and/or subclinical) were compared with euthyroid controls; (5) patients had to be diagnosed based on clinical, biochemical and thyroid profile evaluation; (6) studies must provide method description for IMA estimation with means and S.D. and (7) articles must be written in english. Full texts of the articles were reviewed when the above criteria could not be confirmed by reading titles and abstracts.

The exclusion criteria were as follows: (1) studies with no control group; (2) studies with less sample size (<10 subjects in cases and or controls); (3) studies reported on diseases other than HT and HYT; (4) studies reported on euthyroid autoimmune thyroiditis; (5) IMA levels were only shown by pictograms with no data and (6) non-english articles. If the same group of authors published articles on the same study population, only a recently published article was selected. Two authors reviewed independently, and discrepancies were discussed for a consensus.

### Data extraction and quality assessment

All the articles were thoroughly read and evaluated. Data extraction included: author’s names, year of publication, country, means and S.D. of age and body mass index (BMI), IMA measurement method and units and other study characteristics. A quality score evaluation for studies was done according to Newcastle–Ottawa Scale (NOS) for case-control studies [24]. Quality assessment of studies to a maximum of eight points included three components: selection, comparability and exposure. Higher the score, better the quality.

### Statistical analysis

In studies where HT and/or HYT patient groups were compared with the euthyroid control group, the meta-analysis was performed with individual patient group (experimental group) compared with controls. Sub-group analysis was performed on overt and subclinical types. As we found only one study in SHYT group, we could not conduct a meta-analysis on this. Therefore, in this meta-analysis, we computed the effect size for the following combinations: HT compared with controls and HYT comapred with controls. In addition, we have also compared IMA levels between OHT and SHT groups; HT and HYT groups; pre- and post-treatment levels in HT; and pre- and post-treatment levels in HYT groups.

We calculated the standardized mean difference (SMD) and its 95% confidence interval (CI) as a summary statistic for the difference of IMA level among thyroid patients and healthy controls. Furthermore, we have also performed a meta-analysis of the correlations between IMA and thyroid profile reported in individual studies to obtain a pooled overall correlation coefficient values in HT and HYT groups. The effect size for SMD and pooled correlation coefficient values were presented as a *Z*-score. The *Z*-score with a *P*-value of <0.05 was considered statistically significant.

The between-study heterogeneity was examined by the Cochrane’s Q statistic and expressed as the percentages of *I*^2^. A *P*-value of <0.10 or *I*^2^ statistic of >50% indicated a significant heterogeneity and therefore, a random-effects model was used to compute SMD; otherwise a fixed-effects model was used.

The risk of publication bias in included studies was studied by Begg’s and Egger’s tests in HT and HYT patients. Moreover, to avoid the risk of bias, general search terms were chosen for a guaranteed retrieval for the inclusion of articles reporting IMA levels as a secondary outcome. All comparisons were two-tailed and all analyses were conducted using the Review Manager software version 5.3. The Begg’s and Egger’s tests were performed using comprehensive meta-analysis, version 3. The MedCalc version 16.2.0 was used for the meta-analysis of correlations.

## Results

### Included studies and characteristics

We found 15 studies related to IMA in thyroid diseases [15–23,25–28,37–38]. Non-english articles on IMA in thyroid disorders were not found. One study on serum IMA levels in euthyroid autoimmune thyroiditis [25] and two ‘letter to the editor’ articles [26,27] were excluded from this meta-analysis. No data have been obtained from unpublished studies.

Finally, a total of 12 studies [15–23,28,37–38] fulfilled the selection criteria were included for further analysis, regardless of IMA measurement method and units. All studies, except two [19,28], presented IMA results in absorbance units (ABU). All included studies reported serum IMA concentrations in the healthy control group and in one or more groups of thyroid disorders. Meta-analysis of IMA in HT included 12 studies with a total of 417 patients and 471 controls [15,17,19,20,23,28,37–38]. And, the meta-analysis of IMA in HYT included six studies with a total of 207 patients and 202 controls [16,19–22].

**Figure 1:**
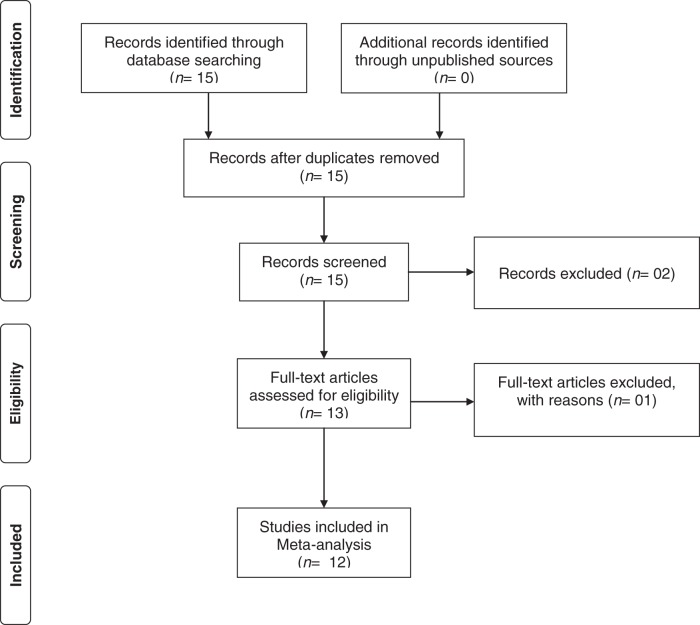
The PRISMA flow diagram

The meta-analysis on pre- and post-treatment IMA values were performed on three independent observations from two studies in HYT group [16,19]. One study contained treatment data on two different groups (overt and subclinical HYT), which were considered independently [16]. Thus, three studies were considered in this comparison. Similarly, the meta-analysis on pre- and post-treatment IMA values were performed on three independent observations from two studies in HT group [19,38]. One study contained treatment data on two different groups (overt and subclinical HT), which were considered independently [38]. Thus, three studies were considered in this comparison. The PRISMA flow diagram is shown in Figure 1. The characteristics and the NOS quality scores of included studies were summarized in Table 1. With the obtained score range from 4 to 8, the overall quality of studies was medium to high.

**Table 1:** Characteristics of included studies in meta-analysis

Study, year/country [Reference]	Controls				Thyroid patients				Study characteristics			
	*N* (M/F)	Mean age ± S.D.	Mean BMI ± S.D.	Thyroid type	*N* (M/F)	Mean age ± S.D.	Mean BMI ± S.D.	Disease duration	Thyroid treatment	IMA method, sample (units)	Matching	NOS
Choudhury et al., 2015/India [37]	43	?	?	HT	56	?	?	?	Not studied	Colorimetric, serum (ABU)	Age and sex	5
Dahiya et al., 2014/India [15]	50 (5/45)	38.1 ± 11.8	22.3 ± 1.9	HT	50 (4/46)	37.9 ± 13.6	32.4 ± 2.1	Newly diagnosed	Not studied	Colorimetric, serum (ABU)	Age and sex	6
Erem et al., 2016/Turkey [38]	30 (9/21)	51.6 ± 17.04	26.53 ± 3.2	HT	30 (6/24)	46.3 ± 14.34	26.33 ± 3.16	3.17	Studied	Colorimetric, serum (ABU)	Age, sex, BMI and BP	8
				SHT	25 (8/17)	47.72 ± 13.6	27.08 ± 3.27	2.92				
Erem et al., 2015/Turkey [16]	30 (9/12)	51.6 ± 17.0	26.5 ± 3.2	HYT	45 (12/33)	50.5 ± 18.0	25.0 ± 3.5	2.4 years	Studied	Colorimetric, serum (ABU)	Age and BMI	8
				SHYT	20 (3/17)	56.1 ± 19.3	26.6 ± 3.6	2.5 years				
Ersoy et al., 2013/Turkey [17]	48 (5/43)	40.0 ± 12.2	30.2 ± 6.6	HT	13 (4/9)	43.2 ± 12.4	29.7 ± 6.5	?	Not studied	Colorimetric, serum (ABU)	Age, BMI, waist circumference and BP	7
				SHT	24 (2/22)	38.0 ± 11.6	28.8 ± 5.7	?				
Lakshminarayana et al., 2014/India [18]	50 (23/27)	?	?	SHT	50	No/Unclear	No/Unclear	?	Not studied	Colorimetric, serum (ABU)	Age and sex matched	4
Ma et al., 2012/China [19]	35 (15/20)	43.0 ± 12.0	24.2 ± 0.9	HT	35 (10/25)	43.0 ± 11.0	25.9 ± 1.4	36 ± 10 months	Studied	Commercial kit, serum (U/ml)	Age, sex and SBP	7
				HYT	35 (13/22)	40 ± 13	23.8 ± 1.1	24 ± 2 months				
Oncel et al., 2014/Turkey [20]	27 (3/24)	38 ± 15.3	No/Unclear	HT	34 (2/32)	37 ± 14.7	No/unclear	Newly diagnosed	Not studied	Colorimetric, serum (ABU)	Age and sex	5
				HYT	27 (4/23)	45 ± 15.7	No/unclear					
Rangaswamy et al., 2014/India [21]	30	?	?	HYT	30	No/Unclear	No/unclear	Newly diagnosed	Not studied	Colorimetric, serum (ABU)	Age and sex	4
Reddy et al., 2015/India [23]	35 (10/25)	33.5 ± 6.8	20.1 ± 1.2	HT	35 (10/25)	33.7 ± 9.7	24.7 ± 3.0	Newly diagnosed	Not studied	Colorimetric, serum (ABU)	Age and BP	7
				SHT	35 (2/33)	33.8 ± 9.4	23.7 ± 3.8					
Roy et al., 2015/India [28]	40	20–45	?	HT and SHT	30	20–45	?	?	Not studied	Colorimetric, serum (IMA Units)	Age and sex	5
Verma et al., 2013/India [22]	50 (4/46)	38.1 ± 11.8	31.2 ± 0.8	HYT	50 (6/44)	44.4 ± 14.4	27.3 ± 0.7	Newly diagnosed	Not studied	Colorimetric, serum (ABU)	Age and sex	6

*N*, total sample size; M: male; F: female; TT3: total tri-iodothyronine; TT4: total tetra-iodothyronine; TgAb: thyroglobulin antibodies; TPOAb: thyroperoxidase antibodies; FT3: free tri-iodothyronine; FT4: free tetra-iodothyronine; BP: blood pressure; SBP: systolic blood pressure; ?: not available/unclear.

### IMA levels in hypothyroid patients

**Figure 2:**
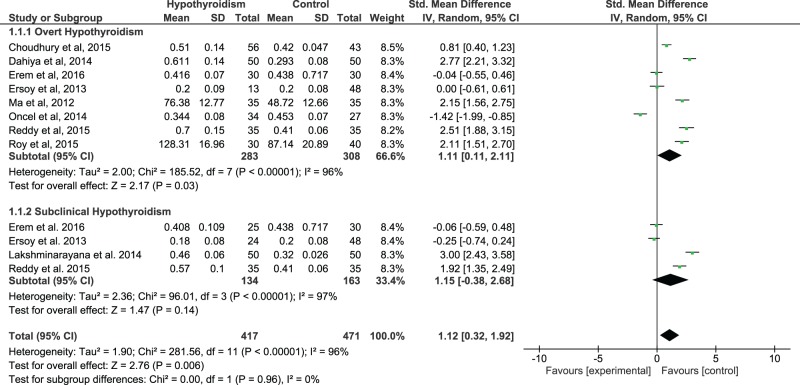
The forest plot of serum IMA levels in hypothyroid patients compared with euthyroid controls

**Figure 3:**
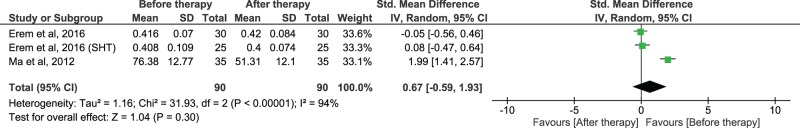
The forest plot of pre- and post-treatment serum IMA levels in hypothyroid patients

As we found a significant between-study heterogeneity (*I*^2^=96%), the random-effects model was applied to compute the pooled effect size. Using all available data to compare serum IMA levels of HT patients compared with controls, there was a significant difference between these two groups. The pooled SMD and (95% CI) were 1.12 (0.32, 1.92). The overall effect size for SMD calculated as *Z* was 2.76 (*P*=0.006), (Figure 2). The sub-group analysis revealed that, in comparison with controls, the serum IMA levels were increased in both overt and subclinical HT groups with statistically significant and non-significant *P*-values (*P*=0.03 and *P*=0.14, Figure 1) respectively. When serum IMA levels in HT patients were compared before and after therapy, a significant heterogeneity was found across studies (*I*^2^=94%), thus the random-effects model was used to compute the pooled effect size. There was no significant difference observed between before and after treatment IMA values. The pooled SMD and (95% CI) were 0.67 (−0.59, 1.93). The overall effect size for SMD calculated as *Z* was 1.04 (*P*=0.30) (Figure 3).

### IMA levels in hyperthyroid patients

**Figure 4:**
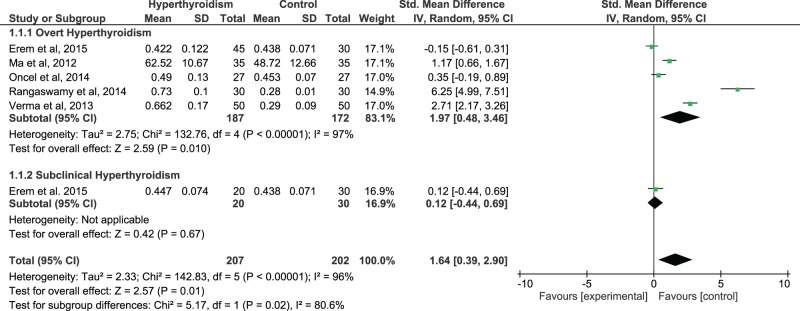
The forest plot of serum IMA levels in hyperthyroid patients compared with euthyroid controls

**Figure 5:**
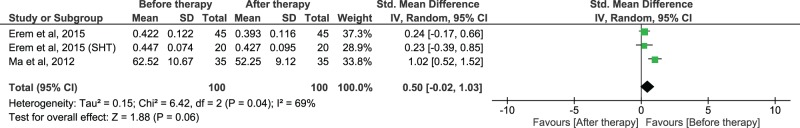
The forest plot of pre- and post-treatment serum IMA levels in hyperthyroid patients

A significant heterogeneity was found across studies (*I*^2^=96%), thus the random-effects model was used to compute the pooled effect size. Using all available data to compare serum IMA levels in HYT patients compared with healthy controls, we found a significant difference between these two groups. The pooled SMD and (95% CI) were 1.64 (0.39, 2.90). The overall effect size for SMD calculated as *Z* was 2.57 (*P*=0.01) (Figure 4). The sub-group analysis revealed that, in comparison with controls, the serum IMA levels were increased in overt HYT group with a statistically significant *P*-value (*P*=0.01; Figure 4), whereas we found only one study with IMA results in subclinical HYT group. When serum IMA levels in HYT patients were compared before and after therapy, a significant heterogeneity was found across studies (*I*^2^=69%), thus the random-effects model was used to compute the pooled effect size. There was no significant difference observed between before and after treatment IMA values. The pooled SMD and (95% CI) were 0.50 (−0.02, 1.03). The overall effect size for SMD calculated as *Z* was 1.88 (*P*=0.06), (Figure 5).

### Publication bias and meta-regression

According to the studies comparing serum IMA levels between HT patients and euthyroid healthy controls, there was no significant publication bias as tested by Begg’s (*P*=0.11) and Egger’s test (*P*=0.27). Also, there was no significant publication bias among the studies comparing HYT patients and euthyroid healthy controls as tested by Begg’s (*P*=0.25) and Egger’s test (*P*=0.08). Sensitivity analysis was performed leaving out one particular study at a time. The results of a leave-one-out meta-analysis showed that no single study had significantly influenced the overall effect size, confirming the reliability and stability of this meta-analysis.

A meta-regression analysis was performed using location and year of the study, age, BMI, sample size and TSH values as covariates. While the study location (coefficient = −1.804; SE = 0.380; *P*=0.001;* P*<0.001) and sample size (coefficient = 0.053; SE = 0.021; *P*=0.010) yielded a statistically significant regression coefficients in HT group, none of these covariates produced significant *P*-values in the HYT group.

### Meta-analysis of correlations between IMA and thyroid profile

The results of this meta-analysis were demonstrated in Table 2. The increased levels of serum IMA in HT patients were found to correlate positively with TSH (pooled *r*=0.48; *Z*=2.84; *P*=0.005) and negatively with TT4 (pooled *r* = −0.39; *Z*= −4.42; *P*<0.001), FT4 (pooled *r* = −0.58; *Z*= −3.27; *P*=0.001) and FT3 (pooled *r* = −0.39; *Z*= −2.17; *P*=0.03). In HYT patients, the increased serum IMA correlated positively with FT4 (pooled *r*=0.40; *Z*=2.57; *P*=0.01) and FT3 (pooled *r*=0.21; *Z*=2.19; *P*=0.02).

**Table 2:** Summary results of a meta-analysis of correlations between serum IMA and thyroid profile in HT and HYT patients

Random effects meta-analysis of correlations in HT							Correlations of IMA with	Random effects meta-analysis of correlations in HYT						
Studies (*n*)	*I*^2^ (%)	Total (*n*)	Pooled (*r*)	95% CI of (*r*)	*Z*	*P*		*P*	*Z*	95% CI of (*r*)	Pooled (*r*)	Total (*n*)	*I*^2^ (%)	Studies (*n*)
7	89.18	291	0.485	0.162 to 0.713	2.838	0.005	**TSH**	0.229	−1.202	−0.436 to 0.111	−0.176	112	53.61	3
3	0.00	120	−0.397	−0.541 to −0.230	−4.426	<0.001	**TT4**	NS	?	?	0.210	50	?	1
1	?	50	−0.330	?	?	NS	**TT3**	NS	?	?	0.036	50	?	1
4	84.29	171	−0.581	−0.787 to −0.260	−3.270	0.001	**FT4**	0.010	2.579	0.102 to 0.636	0.403	112	63.07	3
2	64.96	85	−0.396	−0.663 to −0.0405	−2.170	0.030	**FT3**	0.028	2.196	0.0233 to 0.388	0.213	112	45.43	3

*I*^2^: heterogeneity; *r*: pooled correlation coefficient; *Z*-score: overall effect size for *r*; *P*: statistical significance.

## Discussion

This is the first time that a meta-analysis examines IMA in human HT and HYT. This meta-analysis suggested that IMA levels in serum were higher in both HT and HYT patients than healthy euthyroid controls, which indicated an increased OXS status.

Although the precise mechanism of IMA formation is unclear, processes such as ischaemia, hypoxia, acidosis, membrane disruption, exposure to free iron, copper and free radicals have been proposed to be involved in the formation of IMA. Exposure of normal HSA to free radicals results in a decreased metal-binding capacity of albumin [16,17]. This modified albumin, known as IMA is determined by ACB assay that measures decreased binding capacity of albumin for cobalt [8].

Thyroid hormones are known to regulate mitochondrial respiration and oxidative metabolism, thus may play an important role in the regulation of free radical production and OXS. Therefore, any variations in thyroid hormone status may result in a possible alteration of OXS status. While there are contrary reports in the literature on the status of OXS in HT [17,29], HYT patients are prone to increased free radical generation and OXS due to increased thyroid hormones, accelerated basal metabolic rate and oxidative metabolism [16,30–32].

### Serum IMA in hypothyroidism

Our results of this meta-analysis (Figure 2) showed significantly higher levels of serum IMA in HT patients as compared with euthyroid control counterparts. This rise in serum IMA level, though evident in both overt and subclinical HT groups, statistical significance was noted only in OHT group. This might be due to normal levels of thyroid hormones despite elevated TSH levels in SHT group.

Decreased thyroid hormones and increased TSH have been reported to play a major role in the generation of OXS and hence increased IMA formation [2–6]. Accordingly, there are studies in HT showing significant negative and positive correlations of IMA with thyroid hormones [15,19,23] and TSH [23] respectively. In contrast, Ersoy et al. [17] found no significant associations between IMA and thyroid hormones. In support of the associations between IMA and thyroid hormones, this meta-analysis of correlation coefficients (Table 2) showed a significant positive correlation between increased IMA and elevated TSH levels in HT patients. In addition, there were strong negative correlations of IMA with TT4, FT4 and FT3 in these patients. These results indicate that hormonal disturbances are associated with increased OXS status in HT patients.

Therefore, we also considered to study and report the effect of normalizing thyroid status on serum IMA levels in HT group. Our meta-analysis (Figure 3) on all the available data showed that L-thyroxine treatment may result in the reduction in IMA levels. However, we did not notice a statistically significant effect which might be due to less number of studies reporting contrast findings. In one study, treatment with 50–100 mg of L-thyroxine for 8–10 weeks significantly decreased the serum IMA levels in HT patients [19]. Contrariwise, Erem et al. [38] showed no difference in pre- and post-treatment IMA levels. Therefore, more studies are needed on this aspect to draw a definite conclusion.

Meta-regression showed that country and sample size of the included studies were a significant sources of heterogeneity. One study from China [19] showed highest SMD followed by studies from India and Turkey. And, the SMD has been appeared to be increased with an increase in sample size. The sample size in the studies of Ersoy et al. [17] and Oncel et al. [20] was comparatively less as compared with other studies. Since dyslipidaemia, a characteristic feature in HT [34,35] contributes to OXS and increased IMA formation [33], we also reviewed lipid profile data among studies. While a couple of studies [15,20] did not report lipids, one study [17] showed no data on total cholesterol (TC) and no difference in triglycerides (TG), low-density lipoprotein cholesterol (LDL) and high-density lipoprotein cholesterol (HDL) between cases and controls. There are two studies [19,23] reporting similar findings with increased TC, TG, and LDL in HT patients. However, these studies differed with respect to HDL. Further, in studies by Dahiya et al. [15], Ma et al. [19] and Reddy et al. [23], HT patients had shown significantly higher BMI than their control counterparts. Therefore, these differences in BMI may partly influence serum IMA levels [36]. Therefore, the between-study heterogeneity may be induced by all these differences among included studies. Moreover, different IMA units across studies could also be a relevant factor. While most of the included reported IMA in ABU, two studies [19,28] reported IMA in other units. However, when a meta-analysis was performed, removing these two studies [19,28], there was no significant change in the effect size of IMA, indicating the effect size observed was independent of IMA reporting units.

### Serum IMA in hyperthyroidism

Our results (Figure 4) showed a very strong effect size of serum IMA levels in HYT patients compared with healthy controls and this increased IMA level has been found to be decreased, though of borderline statistical significance (*P*=0.06), after HYT treatment (Figure 5).

Meta-regression revealed that location/country, publication year, sample size, age, BMI and TSH were no significant source of heterogeneity. Therefore, methodological variations such as IMA units, study design, thyroid hormones, and disease duration could possibly attributable to the discrepancy. One study by Ma et al. [19] reported IMA in U/ml whereas other studies reported in ABU. However, a meta-analysis performed after removing the study of Ma et al. [19] showed no significant change in the effect size of IMA, indicating the effect size observed was independent of IMA reporting units. The disease duration and lipid profile might have also contributed to the between-study heterogeneity. While three [20–22] of the included studies do not report lipid profile, the other two studies showed hypolipidemia in HYT patients [16,19]. The disease duration was found to be more than 2 years in HYT patients of two studies [16,19], whereas the other three studies [20–22] included newly diagnosed patients.

Furthermore, the HYT studies included in meta-analysis showed different levels of thyroid hormones (total or free) having different units. Considering the evidence of increased thyroid hormones in inducing OXS, this variation between studies may possibly result in between-study heterogeneity. This is further strengthened by the discrepancy among studies; few studies [19,20] reported significant positive associations between IMA and thyroid hormones, whereas others [16,21,22] do not. It was further reported that increased FT3 as the most significant factor affecting serum IMA levels [19]. In support of the associations of excess thyroid hormones with elevated IMA status, this meta-analysis of correlation coefficients showed strong positive associations of IMA with FT4 and FT3 in HYT patients (Table 2).

Therefore, we further reviewed the effect of treating HYT patients on the serum IMA level (Figure 5). There were two reports [16,19] with a pre-treatment and post-treatment study design. Despite similar medication, these two studies found conflicting results. In a study by Ma et al. [19], treating HYT patients with 20–30 mg of methimazole for 10–12 weeks brought a significant decrease in serum IMA levels. Contrariwise, in a study by Erem et al. [16], treating HYT and SHYT patients with methimazole (initial dose of 15 and 30 mg/day) or PTU (initial dose of 150 and 300 mg/day) for one month to maintain euthyroidism showed no significant change in serum IMA levels. This discrepancy in results may be possibly due to different treatment periods and baseline pre-treatment IMA levels. While Ma et al. [19] found a significantly higher pre-treatment IMA level in HYT patients, Erem et al. [16] reported a no change in the pre-treatment serum IMA level in HYT and SHYT patients compared with euthyroid controls. Although we found in this meta-analysis (Figure 5), a favorable effect of HYT treatment on serum IMA levels, the overall effect size obtained has borderline statistical significance (*P*=0.06). Therefore, more studies with larger sample sizes and similar drug treatment periods are needed.

To our knowledge, the present study is the first of its kind a meta-analysis on the serum IMA level as a simple measure of OXS in human hypothyroidism and hyperthyroidism. Because of the inherent limitations in the observational/case-control study designs and the high possibility of heterogeneity and publication bias in a meta-analysis, we cannot completely exclude the possibility of bias. However, we minimized publication bias by searching major databases with MESH terms and general keywords and also by a quality assessment of each study included by using NOS criteria for case-control studies. Random-effects model was used for SMD estimation for its advantages over the fixed-effect model in the case of between-study heterogeneity. Importantly, there was no publication bias as suggested by Begg’s and Egger test outcomes. The available literature shows less number of studies in a specific type of thyroid dysfunction and scarcity of reports on the effect of thyroid treatment on serum IMA level. Furthermore, most of the studies included in this meta-analysis reported serum IMA as a biomarker of OXS. Its role as a diagnostic and or prognostic marker and clinical utility in association with disease severity and treatment needs to be addressed in future studies.

In summary, our meta-analysis suggests that serum IMA levels increase significantly in both HT and HYT patients as compared with euthyroid controls. The hormonal changes in thyroid dysfunction are associated with the elevated IMA levels. Further, respective thyroid treatment showed to decrease serum IMA levels, prominently in HYT patients. The findings of this meta-analysis recommend the serum IMA as a simple measure of OXS in thyroid dysfunction. More studies in future are needed to definitely conclude the effect of thyroid treatment on serum IMA levels.

## Abbreviations

TERM: Definition
CI: confidence interval
HSA: human serum albumin
HT: hypothyroidism
HYT: hyperthyroidism
IMA: ischaemia-modified albumin
MeSH: medical subject heading
NOS: Newcastle–Ottawa scale
OHT: overt hypothyroid
OHYT: overt hyperthyroid
OXS: oxidative stress
SE: standard error
SHT: subclinical hypothyroid
SHYT: subclinical hyperthyroid
SMD: standardized mean differences
TSH: thyrotropin
